# Chitosan-Coated Mesoporous Silica Nanoparticles Co-Loaded with Curcumin and Amphotericin B: A Drug Delivery Approach for Photodynamic Inhibition of Dual-Species Biofilms

**DOI:** 10.3390/pharmaceutics18060644

**Published:** 2026-05-23

**Authors:** Shima Afrasiabi, Mohammad Reza Karimi, Sepideh Khoee, Stefano Benedicenti, Antonio Signore

**Affiliations:** 1 Laser Research Center of Dentistry, Dentistry Research Institute, Tehran University of Medical Sciences, Tehran 1417614411, Iran; 2 Polymer Laboratory, School of Chemistry, College of Science, University of Tehran, Tehran 1417614411, Iran; r.karimi69@ut.ac.ir (M.R.K.); khoee@ut.ac.ir (S.K.); 3 Department of Surgical Sciences and Integrated Diagnostics, University of Genoa, Viale Benedetto XV, 6, 16132 Genoa, Italy; benedicenti@unige.it; 4 Therapeutic Dentistry Department, Institute of Dentistry, I.M. Sechenov First Moscow State Medical University, Trubetskaya Str. 8, b. 2, 119992 Moscow, Russia; dr.signore@icloud.com

**Keywords:** amphotericin B, biofilms, curcumin, chitosan, drug delivery, natural products, photochemotherapy

## Abstract

**Background/Objectives:** Metabolic dormancy in biofilms leads to reduced drug efficacy in these communities. Different pharmacokinetics and adverse side effects complicate the simultaneous delivery of multiple drugs at appropriate concentrations to the infection site. This study aimed to develop chitosan-coated mesoporous silica nanoparticles loaded with curcumin and amphotericin B (CS@MSNs-Cur-AmB) and to evaluate their antibiofilm activity combined with antimicrobial photodynamic therapy (PDT) against *Streptococcus mutans* and *Candida albicans* dual-species biofilms. **Methods:** CS@MSNs-Cur-AmB were developed. The structure and morphology of the nanoparticles were evaluated using Fourier transform-infrared spectroscopy (FTIR), zeta potential, field emission scanning electron microscopy (FESEM), and thermogravimetric analysis (TGA). Cytotoxicity toward human gingival fibroblasts was assessed. Colony-forming units per milliliter (CFU/mL) were determined. The metabolic activity of biofilm-forming cells was measured using the tetrazolium (MTT) assay. **Results:** Physicochemical analyses confirmed the synthesis of CS@MSNs-Cur-AmB, revealing a particle size of 228 nm and thermal stability up to 600 °C. Cytotoxicity assays showed that CS@MSNs-Cur-AmB exhibited good biocompatibility (>90%). CS@MSNs-Cur-AmB improved antimicrobial activity, which was further enhanced by blue light-emitting diode (LED) irradiation. CS@MSNs-Cur-AmB under LED irradiation showed the strongest effect, reducing metabolic activity to 27.74 ± 4.08% (1 W/cm^2^, 1 min), *p* < 0.001). **Conclusions:** Formulating two drugs in nanocarrier systems may improve therapeutic efficacy by increasing local concentration and reducing systemic exposure. This offers an effective strategy for combating oral biofilms.

## 1. Introduction

The co-existence of *Streptococcus mutans* and *Candida albicans*, together with the production of extracellular polymer substances (EPS), plays a key role in the formation of dental plaque [1]. This structure provides an effective physical barrier to antibiotic penetration and means that the effective concentration of the drug at the biofilm site is insufficient. Consequently, higher drug doses are required, which carries the risk of resistance development [2]. Therefore, the emergence of complementary therapeutic strategies or combinatorial therapies is crucial.

Antimicrobial photodynamic therapy (PDT) involves the interaction of light of appropriate wavelength, a photosensitizer (PS), and molecular oxygen, which kills the target cells by inducing reactive oxygen species (ROS) [3]. PDT is a promising alternative to antibiotics as a treatment modality for localized bacterial elimination in oral infections [4]. Curcumin (Cur) is a natural polyphenol extracted from the rhizomes of *Curcuma longa* and has been extensively studied for its extensive medicinal properties. Cur has been shown to reduce biofilm mass and inhibit EPS matrix formation in *S. mutans* and *C. albicans* biofilms. In addition, Cur significantly downregulates *gtfB* and *gtfC* genes and quorum sensing-related gene expression in *S. mutans*, which are essential for mediating the interaction between *C. albicans* and *S. mutans* [5]. However, the use of Cur is limited due to its poor solubility and instability when exposed to light, meaning that ROS formation and PDT activity are reduced [6].

Several strategies have been investigated to improve the use of Cur, most of which aim to reduce its degradability and increase its solubility. Among these alternatives, encapsulation in controlled delivery systems, such as nanoparticles, is attracting increasing interest [7]. Amphotericin B (AmB) is a fast-acting polyene macrolide antifungal that binds with high preference to ergosterol in the fungal cell membrane. Upon binding, two major pathways for cell death are observed: the formation of channel-like complexes in the lipid bilayer that increase membrane permeability to ions and small molecules and allow leakage of cell contents [8,9]. In addition, the extramembrane organization of the molecules into a “sterol sponge” pulls ergosterol out of the membrane and disrupts membrane function; both mechanisms lead to the breakdown of membrane homeostasis and cell death [10]. The selectivity of AmB is largely due to its preference for fungal ergosterol over mammalian cholesterol, although these membrane interactions are responsible for side effects such as nephrotoxicity [11].

The development of systems for the controlled release of drugs or natural substances is a promising strategy to enhance the therapeutic performance and stability of bioactive agents [12,13]. In this sense, mesoporous silica nanoparticles (MSNs) with biocompatibility and high pore volume are considered promising nanocarriers for drug delivery due to their low cost, diverse synthesis methods, and easy surface modification [14]. The porous silica substrate has pores that can increase the surface area required for loading Cur and AmB, thereby increasing efficacy at lower concentrations. To prevent the release of the transported substances at undesired sites, MSNs can usually be combined with polymer coatings. Chitosan (CS) can be a suitable choice due to its intrinsic antibacterial properties and non-cytotoxic nature [15]. The CS coating on the surface of the nanoparticles imparts antibacterial properties to the nanoparticles. In addition, the CS chains can also provide a site for further loading of Cur. The drug loaded on the surface of these nanoparticles is gradually released into the environment and the duration of drug retention in the environment is prolonged [16].

Although previous studies have reported Cur-based PDT systems [17] or MSN-Cur-based PDT [12], this study focuses on developing CS-coated MSNs co-loaded with Cur and AmB (CS@MSNs-Cur-AmB) for combined antimicrobial and PDT applications. In addition, this study specifically investigates the efficacy of this nanosystem against dual-species biofilms of *S. mutans* and *C. albicans*, which are strongly associated with oral infectious biofilms and the progression of dental caries. In this work, MSNs were synthesized and coated with CS to produce CS@MSNs. Both MSNs and CS@MSNs were characterized by Fourier transform-infrared spectroscopy (FTIR), dynamic light scattering (DLS), surface charge (ζ-potential) measurements, field emission scanning electron microscopy (FESEM), and thermogravimetric analysis (TGA). The effects of the prepared nanoparticles on dual-species biofilms and metabolic activity were also investigated.

## 2. Materials and Methods

### 2.1. Materials

Tetraethyl orthosilicate (TEOS), 25% ammonia solution, ethanol, (3-aminopropyl) triethoxysilane (APTES), low-molecular-weight CS (CAS Number: 9012-76-4) with a molecular weight of 50,000–190,000 Da (based on viscosity) and a deacetylation degree of 75–85%, 1-ethyl-3-(3-dimethylaminopropyl) carbodiimide hydrochloride (EDC. HCl), dimethyl sulfoxide (DMSO), dimethylformamide (DMF), succinic anhydride, triethylamine, N-hydroxysuccinimide (NHS), Cur, dialysis bags with a cut-off of 12 kDa and dodecyltrimethoxysilane, and brain-heart infusion (BHI) broth were all purchased from Merck, Darmstadt, Germany. BHI agar was purchased from QUELAB, Manchester, UK. Sabouraud dextrose (SD) broth and SD agar were obtained from Ibresco, Tehran, Iran. AmB and 3-(4,5-dimethylthiazol-2-yl)-2,5-diphenyltetrazolium bromide (MTT) were supplied from Sigma-Aldrich, Billerica, MA, USA. Human gingival fibroblasts (HGFs) were obtained from Dentistry Research Institute, Tehran University of Medical Sciences, Tehran, Iran.

### 2.2. Instruments

The steps of synthesis were characterized by FTIR (Bruker-Equinox55- Billerica, MA, USA) spectroscopy. To determine the size of the produced particles, two techniques were used: FESEM (FESEM; MIRA3TESCAN-XMU-Brno, Czech Republic) and DLS (HORIBA-SZ100-Kyoto, Japan). FESEM was used to analyze the morphology and size of nanoparticles, as well as to clearly observe particle structure. DLS was used to measure the hydrodynamic particle size distribution, confirming that they are nanosized. The average hydrodynamic diameter, size distribution, and ζ-potential of the aqueous dispersion of the nanoparticles were determined using DLS at 25 °C. The nanoparticles were dispersed in distilled water and a drop of this dispersion was placed on a carbon-coated grid. The grid was completely dried and used for FESEM analysis (10 kV). TGA was performed to indicate the content of the polymer on the surface of the nanoparticles (TGA Q50 V6.3 Build 189). The samples were heated from 20 to 600 °C at a rate of 10 °C/minute under a nitrogen blanket. Drug-loading and drug release were analyzed using an ultraviolet–visible light (UV–Vis) spectrophotometer (Jasco Inc V-750, Tokyo, Japan). Nanoparticles were collected using a centrifuge manufactured in Iran by Pars Azma Company, Isfahan, Iran. The centrifuge was operated at 3000 rpm for 15 min at room temperature to collect and wash nanoparticles at various stages. The freeze-drying system (A-10, Tajhizat Sazan Pishtaz, Tehran, Iran) was used to dry the samples at a temperature of −48 °C and a pressure of 1 mbar.

### 2.3. Synthesis of MSNs via Sol–Gel Method

MSNs were prepared using the sol–gel technique [18]. Initially, 10 mL (0.17 mmol) of ethanol, 3.7 mL (0.20 mmol) of deionized water, and 1.9 mL (0.12 mmol) of a 25% *v*/*v* ammonia solution were added to a round-bottom flask and stirred for 15 min. While stirring the solution with a magnetic stirrer, 1 mL (4.51 mmol) of TEOS and 100 µL of dodecyltrimethoxysilane were added simultaneously. The reaction mixture was shaken continuously for two hours. The synthesized silica was isolated by centrifugation at 3000 rpm for 15 min. The precipitate was washed twice with ethanol and twice with deionized water to remove unreacted solvents, and then freeze-dried. The resulting white silica powder was then calcined at 550 °C for 1.5 h to create porosity. To remove organic residues, the MSNs were rinsed twice with ethanol and then with water, followed by freeze-drying. The final product yield was 92%.

### 2.4. Surface Functionalization of MSNs with Amine Groups

MSNs (400 mg) were dispersed in 120 mL of ethanol using 45% ultrasound for 5 min. The resulting solution was transferred to a three-neck flask with a nitrogen inlet and outlet and shaken at 700 rpm for 15 min. Then, 3 mL (12.82 mmol) of APTES was added, followed by the addition of 2 mL of deionized water. After a period of 10 min, the nitrogen supply was stopped, and the mixture was stirred at room temperature for 6 h. The nanoparticles were isolated by centrifugation, washed twice with ethanol and twice with water, and then freeze-dried [19]. Amine-functionalized spherical MSNs were synthesized with a yield of 85%.

### 2.5. Carboxylic Acid Functionalization of Amine-Modified MSNs

A total of 200 mg of the previously synthesized MSNs was dispersed in 5 mL of anhydrous DMF using ultrasonication for 15 min. Two to three drops of triethylamine were used to activate the amine groups and were swirled for another 15 min. Then, 1.32 g (13.19 mmol) of succinic anhydride was dissolved in 5 mL of anhydrous DMF and introduced at 40 °C. The reaction occurred at 40 °C for 48 h. The nanoparticles were finally isolated by centrifugation, followed by purification with acetone, ethanol, and deionized water, and then freeze-dried [20]. Spherical MSNs functionalized with carboxylic acid were synthesized with a yield of 80%.

### 2.6. CS Coating of MSNs

Carboxyl-functionalized MSNs (100 mg) were suspended in 10 mL of water. Then, 30 mg of EDC and 20 mg of NHS were added and the solution was shaken for 2 h. Subsequently, 200 mg of CS was added and the reaction was allowed to run for 24 h at room temperature. The nanoparticles were isolated by centrifugation, rinsed twice with water, and then freeze-dried.

### 2.7. Drug Loading of Cur and AmB into CS@MSNs

Ten milligrams of CS@MSNs were suspended in 2 mL of DMF, followed by incorporation of 5 mg of Cur and 100 μg of AmB. The solution was then added dropwise to 20 mL of water. The drug-loaded nanoparticles were isolated by centrifugation, rinsed with water, and then freeze-dried. Another batch of nanoparticles was infused with only Cur using the same procedure.

### 2.8. Determination of Drug Entrapment Efficiency in CS@MSNs

To measure drug loading, a certain number of drug-loaded nanoparticles was suspended in 2 mL of DMSO and shaken at 250 rpm for 30 min. The nanoparticles were then isolated by centrifugation, and the UV–Vis absorbance of the supernatant was measured at 360 nm for curcumin and 417 nm for AmB. The drug concentrations were determined using the Beer–Lambert rule. The drug loading capacity and entrapment efficiency were determined using Equations (1) and (2):

Drug entrapment efficiency (%) = (mass of entrapped drug/initial drug mass) × 100(1)

Drug loading capacity (%) = (mass of entrapped drug/mass of nanoparticles) × 100(2)

### 2.9. Examining the In Vitro Drug Release from CS@MSNs

One milligram of the nanoparticle was added to a dialysis membrane with a cut-off of 12 kDa. The release process was carried out in phosphate-buffered saline (PBS) at a pH of 7.4 and a temperature of 37 °C. The drug release from the nanoparticles was measured using the UV–Vis method at 220 nm and 202 nm for Cur and AmB, respectively, and the percentage of drug release was determined using Equation (3) as follows:

Drug release percentage = M_t_/M_0_ × 100 (3)

where M_0_ and M_t_ are the amounts of drug loaded and released, respectively, at time t. Each experiment was performed in triplicate.

### 2.10. Cytotoxicity MTT Assay

HGFs were cultured for 24 h in DMEM, containing 10% fetal bovine serum at a concentration of 10^4^ cells/mL, and then treated with AmB, Cur, CS@MSNs-Cur, and CS@MSNs-Cur-AmB at the desired concentrations. After 24 h of incubation, the culture media were discarded, and 50 μL of MTT (5 mg/mL PBS) was added to each well, followed by incubation for 4 h (37 °C, 95% humidified air, and 5% CO_2_). Then, 150 μL DMSO was added to each well. An ELISA microplate reader with a wavelength of 570 nm was used to measure the results after the plates were shaken for 20 min [21]. Untreated cells were used as control, while DMEM served as blank.

### 2.11. Minimum Inhibitory Concentration

The minimum inhibitory concentration (MIC) of CS@MSNs-Cur-AmB was determined against the test strains according to Clinical Laboratory Standards Institute (CLSI) methods [22]. In brief, overnight grown cultures of *S. mutans* IBRC-M 10,682 and *C. albicans* ATCC 10,231 were prepared in BHI broth and SD broth, respectively. Furthermore, 50 μL of *S. mutans* inoculum (5 × 10^5^ CFU/mL), 50 μL of *C. albicans* (5 × 10^3^ CFU/mL), and 0.1 mL of twofold serial dilution of CS@MSNs-Cur-AmB were added to each well of a 96-well microtiter plate. A control test was performed without any antimicrobial agent. The MIC was defined as the lowest concentration of antimicrobial agent that inhibited visible growth of the test organism.

### 2.12. Antimicrobial Assay

*S. mutans* and *C. albicans* were grown overnight at 37 °C in 5 mL BHI broth and SD broth, respectively. Fifty microliters each of *S. mutans* (5 × 10^6^ CFU/mL) and 50 μL each of *C. albicans* (5 × 10^5^ CFU/mL) were added to a 96-well microplate. To establish dual-species biofilms, mixed microbial suspensions were incubated for 48 h at 37 °C and 5% CO_2_ to allow biofilm formation and maturation. After incubation, the wells were gently washed with sterile PBS to remove non-adherent cells before treatment application. For treatment, 100 μL of CS@MSNs-Cur-AmB at sub-MIC concentration was added to the preformed biofilms. After a 5 min incubation in a dark room at 25 °C, the diluted bacterial suspension (10 μL) was spread evenly onto BHI agar supplemented with AmB (select for *S. mutans*) and SD agar supplemented with chloramphenicol (select for *C. albicans*). BHI agar plates were incubated for 48 h at 37 °C and 5% CO_2_ for CFU (cells/mL) enumeration. In addition, SD agar plates were incubated aerobically for 24 h at 37 °C. The same procedure was performed to evaluate the antimicrobial activity of free AmB, free Cur, and CS@MSNs-Cur at concentrations equivalent to those present in the final CS@MSNs-Cur-AmB suspension. To evaluate the photodynamic effects of free Cur, CS@MSNs-Cur, and CS@MSNs-Cur-AmB on the viability of the strains, the experiments were performed by exposing each suspension in the same manner as described above, but with a light-emitting diode (LED) (430–460 nm, 1 W/cm^2^, 60 s). The LED source was positioned at a fixed distance of 1 mm above the sample surface to ensure uniform irradiation. During irradiation, adjacent wells were covered with aluminum foil to prevent unintended light exposure. The temperature was monitored throughout the irradiation process, and no significant increase was observed under the experimental conditions.

### 2.13. Colorimetric Assay

The MTT assay was used to verify the effects of the different treatments on the viability of the biofilms. For the MTT assay, 50 μL of 5 mg/mL MTT was added to the wells and incubated at 37 °C for 4 h in the dark. Then, 150 μL DMSO was added and shaken for 10 min. The samples were analyzed using a microplate reader and the absorbance at 570 nm was recorded.

### 2.14. Statistical Analysis

All data are given as mean values ± standard deviation (SD). The statistical analyses were carried out using IBM SPSS Statistics version 26.0 (IBM Corp., Armonk, NY, USA). Statistical analyses of the data were performed using one-way analysis of variance (ANOVA) with post hoc test (Tukey’s multiple comparison) and significant difference was considered to be *p* < 0.05.

## 3. Results and Discussion

### 3.1. Characterization of MSNs

**Figure 1:**
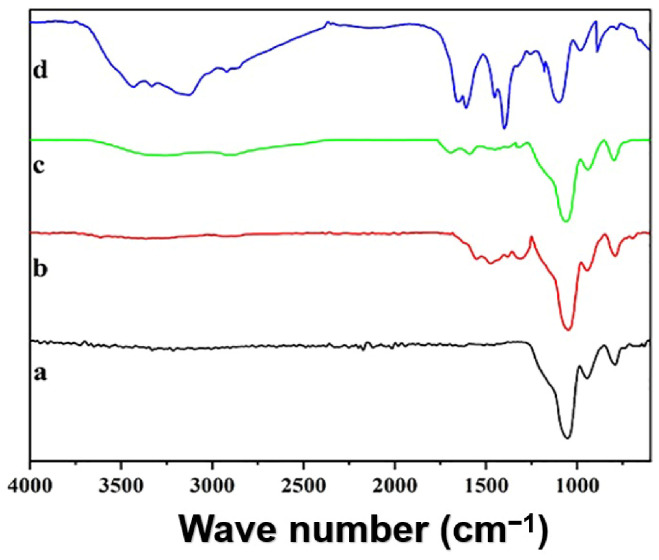
FTIR spectra of (**a**) MSNs, (**b**) amine-functionalized MSNs, (**c**) carboxylic acid-functionalized MSNs, and (**d**) CS@MSNs.

The MSNs were prepared using the sol–gel method, and their surfaces were functionalized with amine and then carboxylic acid groups. CS was conjugated by amide bond formation between its amine groups and the carboxyl groups on the silica surface. In the FTIR spectra of MSNs (Figure 1a), the Si-O stretching peak was seen at around 1100 cm^−1^. The FTIR spectrum of amine-functionalized particles showed distinct peaks at around 2900 cm^−1^ (C-H), 1548 cm^−1^ (N-H bending), 1466 cm^−1^ (C-N), and a broad peak in the range of 3000–3500 cm^−1^ (N-H stretching), confirming amine functionalization (Figure 1b). For carboxylic acid-functionalized particles, C=O stretching was observed at around 1600 cm^−1^ for amide groups and around 1700 cm^−1^ for carboxylic acid groups. The N-H stretches coincided with the O-H stretches at around 3400 cm^−1^. The Si-O peaks remained prominent (Figure 1c). The FTIR analysis of CS@MSNs revealed a conspicuous amide bond at around 1625 cm^−1^ and N-H bending at around 1556 cm^−1^, confirming the presence of CS. The absence of a signal at ~1700 cm^−1^ indicates the successful interaction between the amine groups of CS and the carboxyl groups of the MSNs (Figure 1d). Intensified peaks at around 1100 cm^−1^ (Si-O), 3300 cm^−1^ (N-H), and 2900 cm^−1^ (C-H) confirmed the presence of CS coating.

**Figure 2:**
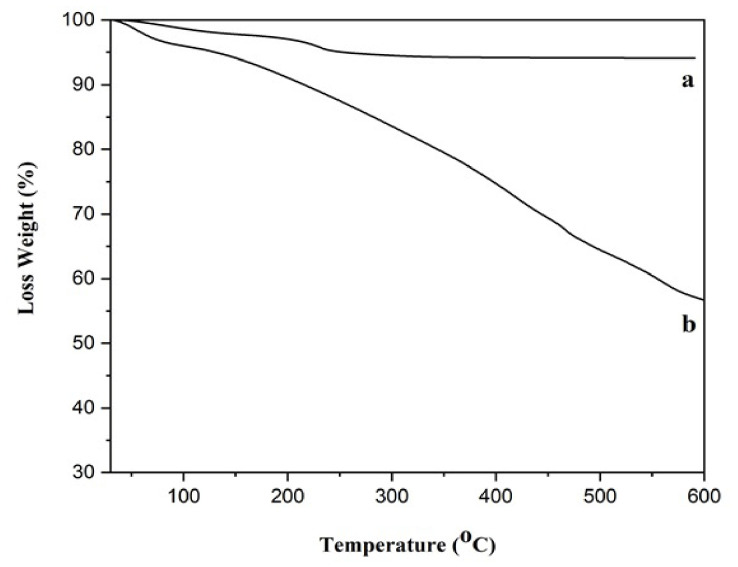

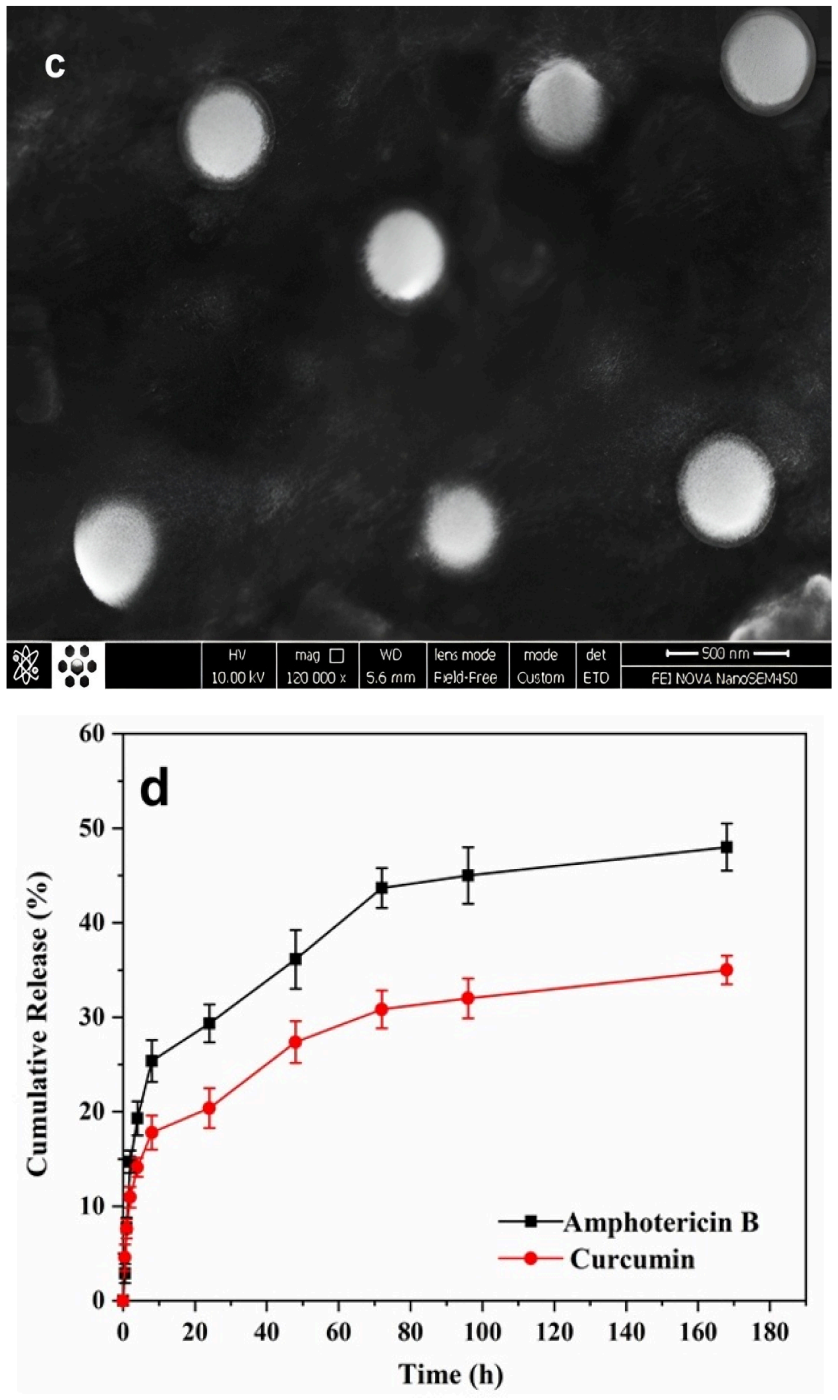
TGA curves of (**a**) MSNs and (**b**) CS@MSNs. (**c**) FESEM image of CS@MSNs. (**d**) The representative release profile of CS@MSNs-Cur-AmB.

TGA was performed to evaluate the amount of CS coating. Figure 2a shows that the uncoated MSNs experienced weight loss of 5% up to 600 °C, which is due to surface water, while CS@MSNs experienced a weight loss of 45%, indicating significant CS coating (Figure 2b). Figure 2c shows the FESEM image of the CS@MSNs, which have a homogeneous spherical shape with a diameter of about 200 nm and a uniform surface coating.

The release profile of drugs from nanoparticles was investigated at pH 7.4. It was found that AmB was more easily dissolved and released in the buffer, due to the smaller amount loaded on the nanoparticles; 48% of the loaded drug was released within a week. Conversely, a larger amount of Cur was encapsulated in the CS@MSNs, resulting in delayed release due to the limited solubility of the drug in the buffer (Figure 2d).

The *ζ*-potential and particle size of the nanoparticles were determined using DLS, and the results are shown in Table 1. The uncoated MSNs showed a surface charge of −17.4 mV. After functionalization with amine groups, the surface charge increased to +14 mV. After functionalization with carboxylic acid, the surface charge decreased to −13 mV. The CS coating on MSNs finally resulted in an increase in the surface charge to +37.1 mV due to the presence of amine groups. The DLS study showed that the hydrodynamic diameter of the final nanoparticles was 228 nm. The particle size increased gradually in each phase, with the size increasing significantly after applying the CS coating due to its higher molecular weight.

### 3.2. Drug Loading and Entrapment Efficiency of Cur and AmB

The drug loading and entrapment efficiency were determined using UV–Vis analysis. The results for Cur were 30.67% and 92%, while for AmB, they were 0.64% and 96%. The hydrophobic nature of the drugs facilitated their interaction with the polymer chains, resulting in increased encapsulation efficiency in the nanoparticles.

### 3.3. Cytotoxicity Assay

**Figure 3:**
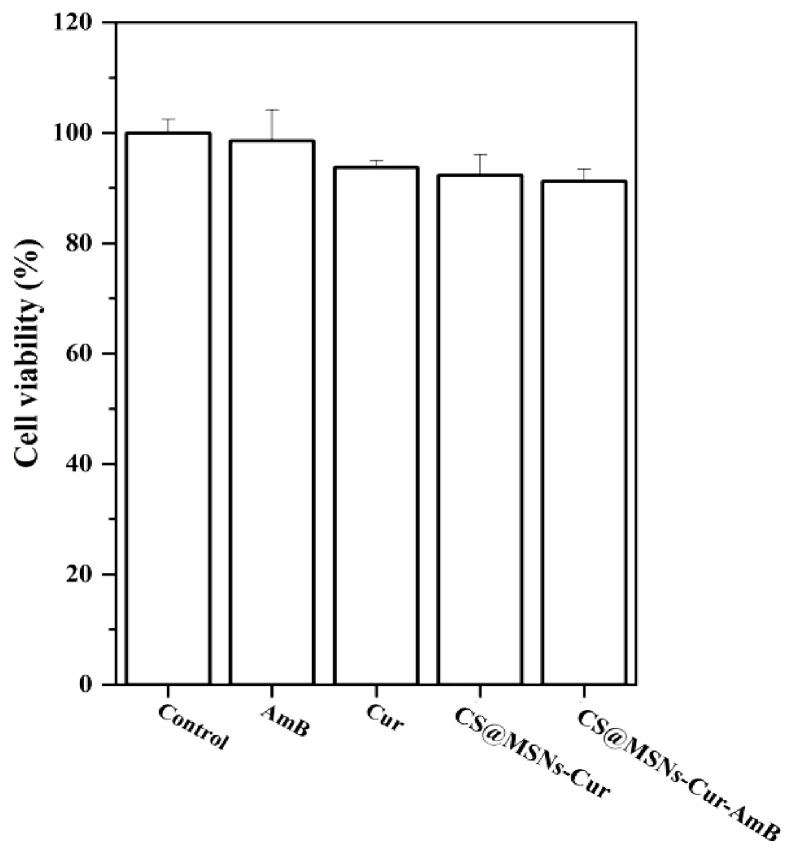
Cytotoxicity of free AmB, free Cur, CS@MSNs-Cur, and CS@MSNs-Cur-AmB on human gingival fibroblasts assessed by MTT assay after 24 h incubation. Data represent mean ± SD (*n* = 3). AmB: amphotericin B, Cur: curcumin, CS: chitosan, MSNs: mesoporous silica nanoparticles.

The MTT assay was used to assess the viability of HGFs after exposure to free AmB, free Cur, or Cur nanoformulations at sub-MIC concentrations. As shown in Figure 3, there was no noticeable effect on HGFs after 24 h of exposure to the different treatments, and cell viability remained above 90%. This is consistent with the main property of the PS, which limited dark toxicity. MSNs are considered biocompatible, as they have shown no cytotoxicity in many cases both in vitro and in vivo, even at relatively high concentrations (up to 1000 μg/mL for 12–24) [23]. Long-term evaluations in mice (LD50 > 1000 mg/kg) have shown no mortality or overt toxicity over 14 days from MSNs at doses of 20–80 mg/kg. Therefore, they are generally described as safe [24]. In the present study, concentrations of Cur and AmB in the sub-MIC range were used that are much lower than the concentrations shown to cause cytotoxicity in previous studies. Several reports have shown that AmB is nephrotoxic in a dose-dependent manner, with the risk of nephrotoxicity increasing at doses above approximately 1 mg/kg in mouse models [25]. In contrast, liposomal forms of AmB have shown less toxicity [26]. In addition, in vitro studies have reported that Cur at concentrations of 50 µM and higher can reduce cell viability and induce apoptosis in various cell lines [27]. Therefore, the lack of toxicity observed in our work is likely due to the use of lower concentrations.

### 3.4. Antimicrobial Activity

The accumulation of *S. mutans* triggers *C. albicans* biofilm formation under in vitro conditions and may satisfy the requirement of metabolites or growth-stimulating factors in mixed biofilm conditions [2]. The metabolic interactions are such that streptococci provide a carbon source for the growth of *C. albicans* by producing lactic acid, which helps to reduce oxygen concentration and create favorable conditions for streptococci [28]. In addition, glycosyltransferase enzymes secreted by *S. mutans* bind to the cell surface of *C. albicans* and facilitate the conversion of sucrose to EPS, which in turn provides attachment sites for *S. mutans*. This unusual interaction increases the microbial load, acidic conditions, and EPS production, which promotes adhesion between the two microorganisms [29]. In streptococci also produce a cell wall-anchored protein that assists in the binding of *Candida* cells. *C. albicans* further utilizes the metabolized products and stimulate the production of an ample amount of EPS, which is important for aggregation and accumulation of *S. mutans* cells to develop mixed biofilms. Additionally, the increased population of *Candida* cells may also reduce the diversity of oral microbiome and substitute the microbial community with streptococci. The synergistic interaction between these pathogens helps in the establishment and pathogenicity in the oral environment [2]. A study reported that *C. albicans* increases enamel demineralization by increasing the cariogenic potential of *S. mutans* [30]. *C. albicans* has several characteristics that contribute to causing dental caries. *C. albicans* has the ability to adhere to hydroxyapatite-like surfaces and cooperate with pioneer bacteria to create a strong bond on the tooth surface. Although it is unable to completely decompose sucrose due to the lack of the enzyme α-glucosidase, it can metabolize glucose, fructose, or lactose and, by producing acid, causes a decrease in pH and the destruction of tooth enamel. It has high tolerance to acidic conditions, which is caused by the activity of proton pumps and H^+^-ATPase. It is able to survive in very acidic environments and, in some cases, is stronger than *S. mutans*. The most important cariogenic feature of this fungus is its interspecies networks with different bacteria, which leads to increased pathogenicity and severity of infection [31].

**Figure 4:**
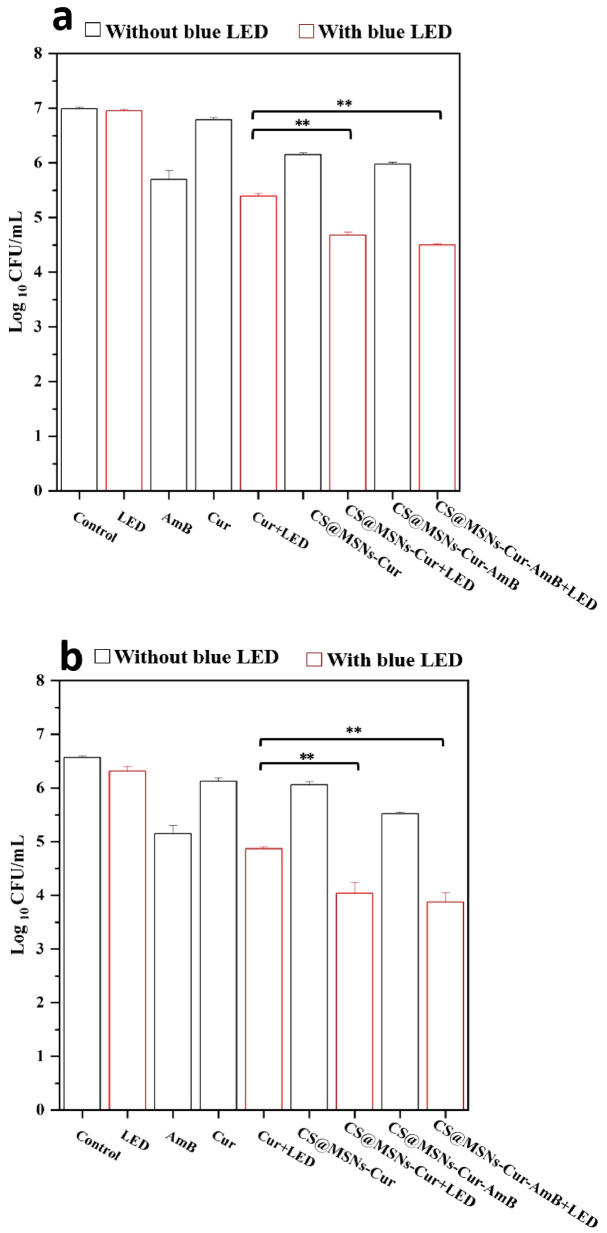
(**a**) Quantitative log_10_ CFU/mL bar graphs of *S. mutans* and (**b**) *C. albicans* after different treatments with AmB, Cur, CS@MSNs-Cur, and CS@MSNs-Cur-AmB, with or without blue LED. Data are presented as mean ± SD (*n* = 3). ** *p* < 0.001. LED: light-emitting diode, AmB: amphotericin B, Cur: curcumin, CS: chitosan, MSNs: mesoporous silica nanoparticles.

In this study, the antimicrobial effect of different formulations, including AmB, Cur, CS@MSNs-Cur, and CS@MSNs-Cur-AmB, with and without blue LED against *S. mutans* and *C. albicans* dual-species biofilms was investigated (Figure 4a,b). Blue LED alone produced only a small reduction in the log_10_ CFU/mL, which was statistically insignificant (*p* = 0.99 and 0.19, respectively). This finding is consistent with previous results showing that LED without a PS has no lethal effect and produces only minor changes in membrane permeability [32]. Therefore, the main efficacy of PDT is entirely dependent on the presence of an active PS [33]. AmB caused a significant reduction in the log_10_ CFU/mL in both microbial models (*p* < 0.001). This result is consistent with its known mechanism, namely, binding to ergosterol in the fungal membrane and creating membrane pores that cause leakage of cell contents and cell death. Although *S. mutans* is a Gram-positive bacterium lacking ergosterol, data indicate its relative sensitivity to AmB [34]. Recent studies have reported that AmB, in addition to its classical effect, can also act by inducing oxidative stress, reducing membrane stability, and disrupting energy metabolism [35,36]. The present results also confirm these observations. However, it seems that AmB alone is not capable of causing a severe or complete reduction in microbial populations, which reinforces the need for combination strategies [37]. Free Cur in the absence of blue LED induced only a very slight reduction in microbial load (less than 0.5 Log_10_ CFU/mL). This finding is consistent with the physicochemical nature of Cur. However, light irradiation caused a dramatic change. This sudden jump indicates the activation of the photodynamic mechanism of Cur. Loading Cur into CS@MSNs resulted in an improvement in its performance, with CS@MSNs-Cur producing a lower Log_10_ CFU/mL than free Cur. In addition, exposure of CS@MSNs-Cur to blue LED resulted in more effective activation of Cur. This finding suggests that the CS@MSNs not only plays a protective role and increases stability, but also provides a suitable substrate for enhancing the photodynamic effect [12]. The combination of Cur and AmB on CS@MSNs showed a greater antimicrobial effect than either component alone in the absence of LED. This suggests an improved interaction between the two active agents. Cur may enhance its efficacy through initial membrane disruption or by facilitating AmB penetration. However, in the presence of LED, the behavior of the system was significantly different. The CS@MSNs-Cur-AmB + LED group showed the greatest reduction in Log_10_ CFU/mL of all the groups. The log_10_ CFU count of *S. mutans* and *C. albicans* cells was reduced from 6.99 to 4.50 log_10_ CFU/mL and from 6.57 to 3.88 log_10_ CFU/mL, respectively, which is greater than the combined effects of Cur + LED and AmB. The disruption of the cell membrane by AmB creates conditions for better penetration of Cur. The simultaneous presence of drug and PS on a nanocarrier increases the local concentration. The inhibition of Cur degradation by the CS@MSNs and the enhancement of oxidative effects in the cell, which were previously attenuated by AmB, can be attributed to this combined effect. This pattern is fully consistent with recent studies in the field of multi-agent nanocarrier systems, showing that the simultaneous use of photodynamic agents and drugs significantly increases the efficacy of treatment [12,37,38].

### 3.5. Anti-Metabolic Activity

**Figure 5:**
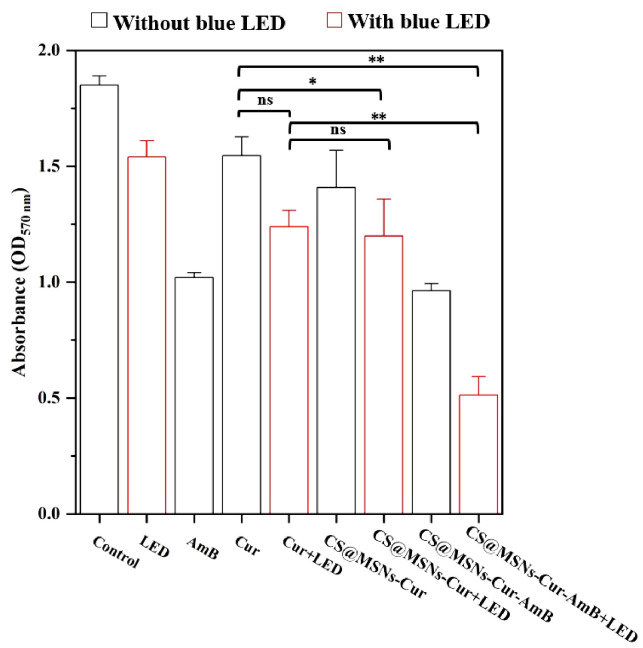
MTT assay for evaluating anti-metabolic effects AmB, Cur, CS@MSNs-Cur, and CS@MSNs-Cur-AmB with or without blue LED. Data are presented as mean ± SD (*n* = 3). ns: not significant, * *p* < 0.05, ** *p* < 0.001. LED: light-emitting diode, AmB: amphotericin B, Cur: curcumin, CS: chitosan, MSNs: mesoporous silica nanoparticles.

The anti-metabolic effects of the experimental groups were evaluated using the MTT assay. As shown in Figure 5, after treatment with Cur, the metabolic activity of dual-species biofilms was 83.58 ± 4.37% (without LED, *p* = 0.12) and 66.94 ± 3.67% (1 W/cm^2^, 1 min), respectively. The metabolic activity in the LED-treated group was 83.25 ± 3.70 (*p* = 0.12), indicating that LED irradiation alone did not produce a significant anti-metabolic effect. Without LED irradiation, the metabolic activity in the CS@MSNs-Cur and CS@MSNs-Cur-AmB groups were 76.12 ± 9.10% (*p* = 0.007) and 52.02 ± 1.56% (*p* < 0.001), respectively, while after irradiation, the metabolic activity decreased to 64.78 ± 8.73% and 27.74 ± 4.08%, respectively (both *p* < 0.001). In addition, AmB exhibited a bacteriostatic effect at a sub-MIC concentration (metabolic activity = 55.16 ± 0.98%, *p* < 0.001). Pairwise comparisons showed that Cur + LED did not significantly differ from Cur alone (*p* = 0.086). However, incorporating Cur into CS@MSNs and applying LED irradiation significantly increased the difference (*p* = 0.038). The most significant difference compared to Cur was observed for photoactivated CS@MSNs-Cur-AmB (*p* < 0.001), indicating that LED is most effective when Cur is incorporated into a combined nanoformulation. Furthermore, Cur + LED showed no significant difference compared to MSN-Cur + LED (*p* = 1.00), but showed a significant difference compared to CS@MSNs-Cur-AmB + LED (*p* < 0.001).

Nanoparticles typically retain the drug as a reservoir and release it gradually. Therefore, during the short duration of the CFU test, the concentration of free drug in the culture medium decreases, and the rapid killing effect is not observed as quickly as with the free form. Previous studies on solid lipid nanoparticles have also reported this issue. The onset of antimicrobial effect in vitro was slower than that of free drugs [39]. The MTT assay shows the difference in the effects of free and nanoparticle forms of drugs on bacterial metabolic activity. It is clear that free AmB has a faster and stronger effect than nanoforms containing Cur, likely due to the immediate availability and high concentration of the free drug in the culture medium. In contrast, CS@MSNs do not have such a strong immediate effect because the gradual release of the drug means that reducing bacterial metabolism requires more time. However, LED irradiation enhanced the antibacterial effect of nanoforms, indicating the important role of Cur photosensitivity. Cur under LED irradiation can produce ROS that cause membrane damage and bacterial cell death. The improved photodynamic effect of Cur and the mechanism of AmB binding to sterols in the microorganism membrane could explain the higher potency of the nanoformulation compared to the free drug. Metabolic assays, which usually depend on cellular activity and metabolism and may take longer, require a gradual release of the drug sufficient to reduce bacterial metabolism [40].

## 4. Conclusions

In this study, we developed CS@MSNs with a uniform particle size distribution and efficiently loaded them with sub-MIC concentrations of Cur and AmB. The nanoparticles were characterized using DLS, zeta potential, FESEM, FTIR, and TGA. The antimicrobial and anti-metabolic effects of Cur and AmB, alone or in combination, were studied with or without irradiation by blue LED light at 430–460 nm (1 W/cm^2^, 60 s) on *S. mutans* and *C. albicans* dual-species biofilms. CS@MSNs-Cur-AmB exhibited prominent antibacterial activity against dual-species biofilms of *S. mutans* and *C. albicans* due to the enhanced effects of PDT. Delivering conventional antibiotics through nanoparticle-based systems offers advantages over free antibiotics, such as reduced concentration and sustained drug release, which contribute to increased antibacterial efficiency. Despite their antimicrobial efficacy, these materials demonstrated a promising safety profile with no indication of cytotoxicity. Such nanocarriers can serve as suitable vehicles for therapies requiring more than one active ingredient.

## Figures and Tables

**Table 1:** Particle size, *ζ*-potential, and polydispersity index (PI) at different synthesis stages.

Sample Name	Size (nm)	Zeta (mV)	PI
MSNs	180	−17.4	0.3
MSNs-NH_2_	189	+14	0.2
MSNs-COOH	194	−13	0.4
CS@MSNs	228	+37.1	0.5

MSNs: mesoporous silica nanoparticles, MSNs-NH_2_: amine-functionalized MSNs, MSNs-COOH: carboxylic acid-functionalized MSNs, CS: chitosan, nm: nanometer, mV: millivolt.

## Data Availability

The original contributions presented in this study are included in the article. Further inquiries can be directed to the corresponding author.
